# Agénésie de la vésicule biliaire: à propos de trois cas

**DOI:** 10.11604/pamj.2017.28.114.11919

**Published:** 2017-10-06

**Authors:** Amine Chouchaine, Mahmoud Fodha, Mohamed Taha Abdelkefi, Kamel Helali, Mohamed Fodha

**Affiliations:** 1 Service de Chirurgie Générale, Hôpital Taher Sfar, Mahdia, Tunisie

**Keywords:** Vésicule biliaire, agénésie, imagerie de la vésicule biliaire, Gallbladder, agenesis, gallbladder imaging

## Abstract

L’agénésie de la vésicule biliaire est une anomalie congénitale rare. Le but de ce travail est d’étudier, à travers trois cas que nous rapportons, les aspects épidémiologiques de cette affection ainsi que les particularités de diagnostic et de prise en charge thérapeutique. Les 2 adultes ont été admis pour coliques hépatiques et troubles dyspeptiques avec à l’échographie une vésicule scléroatrophique multilithiasique. L’un des deux a eu un scanner qui a objectivé l’aspect d’un calcul au niveau d’une vésicule scléroatrophique. Ces 2 malades ont été opéréspar voie classique à tort pour lithiase vésiculaire. L’absence de vésicule biliaire a été découverte en per opératoire. Afin de confirmer le diagnostic en post opératoire, on a pratiqué une bili-IRM pour la 1ère patiente. L’autre malade a été perdu de vue. Notre 3^ème^ cas, une enfant de 13 ans, hospitalisée pour pancréatite aigüe et l’agénésie vésiculaire a été suspectée devant l’aspect scannographique, puis confirmée par la Bili-IRM et elle n’a pas été opérée.

## Abstract (en)

Gallbladder agenesis is a rare congenital anomaly. This study aimed to highlight the epidemiological aspects of this condition as well as the peculiarities of its diagnostic and therapeutic management through three case reports. Two adults were admitted to Hospital with hepatic colics and dyspepsia. Ultrasound showed multilithiasic scleroatrophic vesicle. In one of the two patients, CT scan results showed a stone at the level of scleroatrophic vesicle. These two patients were wrongly operated for vesicular lithiasis by using conventional method. The absence of gallbladder was detected during surgery. In order to confirm post-operative diagnosis, the first patient underwent biliary MRI. The other patient was lost to follow-up. The third patient was a 13-year old child hospitalized with acute pancreatitis. Vesicular agenesis was suspected based on its scannographic aspect and then confirmed using biliary MRI. This patient didn’t underwent surgery.

## Introduction

L’agénésie de la vésicule biliaire (AVB) est définie par l’absence congénitale de la vésicule biliaire (VB) associée ou non à l’absence du canal cystique. C’est une affection congénitale rare du système biliaire.Près de 400 cas d’AVB cliniques et autopsiques ont été rapportés dans la littérature [1]. L’agénésie étant attribuée à une anomalie de développement embryonnaire de mécanisme très probablement génétique, la plupart des cas d’AVB sont associés à d’autres anomalies congénitales. Ces dernières sont présentes essentiellement à la naissanceet la très grande majorité d’entre elles est létale durant la première année de vie. Ce qui fait qu’on ne voit que des patients ayant une AVB isolée à l’âge adulte, ou quelquefois associée à des anomalies des voies biliaires tel que le kyste du cholédoque [2]. Nous rapportons, à travers cette étude de trois cas colligés au service de chirurgie de l’hôpital Taher Sfar de Mahdia, les aspects épidémiologiques de l’AVB ainsi que les particularités du diagnostic et de prise en charge thérapeutique.

## Patient et observation

### Observation n° 1

**Figure 1:**
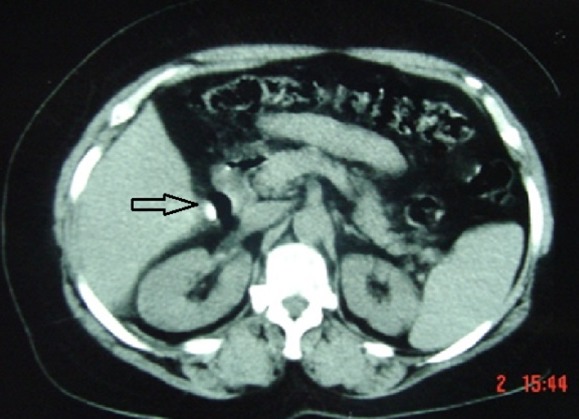
Coupe axiale tomodensitométrique montrant une image de densité calcique se projetant au niveau de l’aire vésiculaire

**Figure 2:**
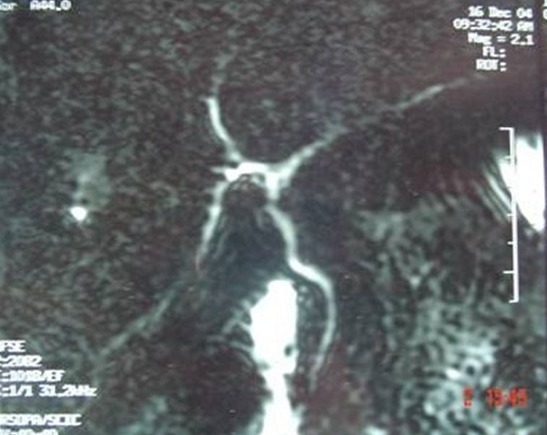
Cholangio-IRM montrant l’absence de vésicule biliaire avec absence de canal cystique

Mme A.Z âgée de 49 ans aux antécédents d’hypertension artérielle, est hospitalisée pour cure chirurgicale d’une lithiase vésiculaire symptomatique. La patiente présente des coliques hépatiques évoluant depuis un an. Le bilan biologique est normal, en particulier pas de cholestase: bilirubine totale et conjuguée, phosphatase alcaline et gamma glutamyl transférase sont normales, il n’y a pas de cytolyse hépatique. Une première échographie abdominale n’a pas visualisé de vésicule biliaire. Un complément d’exploration par un scanner abdominal objective une image calcique se projetant au niveau de l’aire vésiculaire interprétée comme un calcul au niveau d’une vésicule scléroatrophique (Figure 1). Le radiologue juge nécessaire de refaire une deuxième échographie concomitante au scanner, qui objective un aspect de vésicule scléroatrophique multi lithiasique avec un gros calcul mesurant 18 mm au niveau du fond vésiculaire, sans dilatation associée des voies biliaires intra et extra hépatiques. Vu l’aspect de vésicule scléroatrophique, on sursoit à la cholécystectomie par voie coelioscopique et on pose l’indication de cholécystectomie par voie sous-costale droite. L’exploration peropératoire trouve des adhérences colo-hépatiques et pariéto-hépatiques en rapport avec une péri hépatite qui sont libérées. Le pédicule hépatique est bien individualisé sans pouvoir visualiser la vésicule biliaire dans sa position habituelle, ainsi qu’après examen de certains sites d’ectopie vésiculaire. La calcification sus décrite sur le scanner n’a pas été retrouvée. L’échographie per-opératoire confirme l’absence de vésicule biliaire ectopique intra-hépatique. On n’a pas réalisé de cholangiographie (CPO) de façon délibérée. Les suites opératoires ont été simples et la patiente a été mise sortante le 3^ème^ jour post-opératoire. La patiente a été suivie à la consultation et n’a plus signalé de douleurs de l’hypochondre droit, qui sont attribuées très probablement aux adhérences péri hépatiques libérées. Afin de confirmer le diagnostic, elle a eu une cholangio-IRM, un mois et demi après l’intervention ayant conclu à une agénésie de la vésicule biliaire avec absence de canal cystique (Figure 2). On n’a pas trouvé d’anomalies biliaires associées chez notre patiente. Elle est restée asymptomatique jusqu’à présent avec un recul de 3 ans.

### Observation n° 2

**Figure 3:**
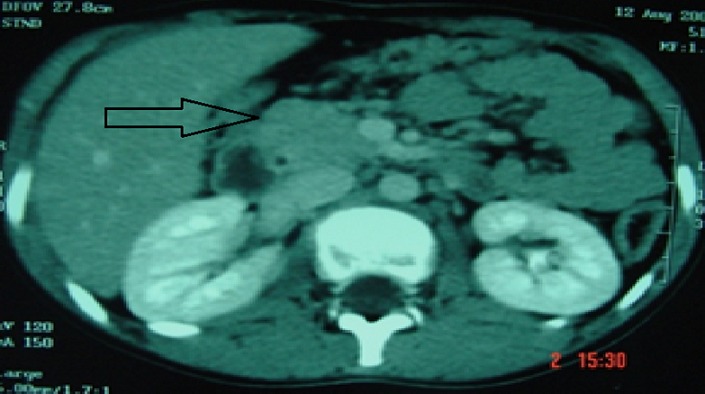
Coupe axiale TDM aspect de pancréatite stade B, et d’agénésie de la vésicule biliaire

**Figure 4:**
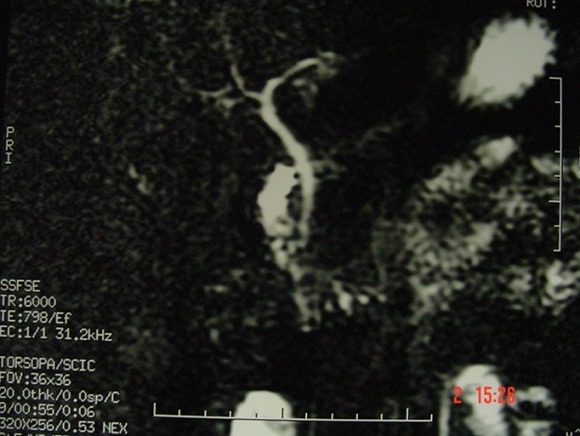
Cholangio-IRM montrant l’absence de vésicule biliaire chez un enfant, sans autres anomalies biliaires associées

Enfant âgée de 13 ans se présente pour des douleurs épigastriques continues sans irradiations particulières avec des vomissements. Dans les antécédents on a noté un rhumatisme articulaire aigu sous Extencilline et une hépatite virale A à l’âge de 10 ans. A l’admission dans le service, température à 37,8°C, la palpation a révélé une sensibilité de la région épigastrique. Les examens biologiques ont objectivé une hyperamylasémie à 5 fois la normale, absence d’hyperleucocytose. Devant ce tableau une pancréatite aigüe est suspectée, pour confirmer ce diagnostic un scanner abdominal a été demandé d’emblée montrant un pancréas globuleux cadrant avec une pancréatite stade B et un aspect d’agénésie de la vésicule biliaire (Figure 3). L’évolution sous traitement médical est rapidement favorable. Au cours du suivi à la consultation externe réapparition de douleurs épigastriques calmées par les repas avec amylasémie normale, la fibroscopie gastrique objective une gastrite nodulaire avec présence d’un petit ulcère au niveau de la grande courbure antrale, la biopsie avec examen anatomopathologique révèle une gastrite chronique antro-fundique folliculaire modérée, légèrement active, avec présence d’Helicobacter Pylori. L’enfant est mis sous trithérapie antiulcéreuse, l’évolution est marquée par la disparition des douleurs. L‘enfant a bénéficié d’une cholangio-IRM concluant à une agénésie de la vésicule biliaire avec absence du canal cystique et sans autres anomalies biliaires associées (Figure 4).

### Observation n° 3

**Figure 5:**
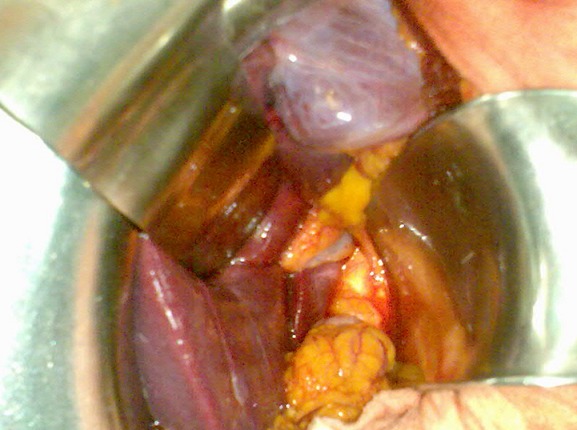
Image per-opératoire montrant l’absence de vésicule biliaire dans sa position habituelle

**Figure 6:**
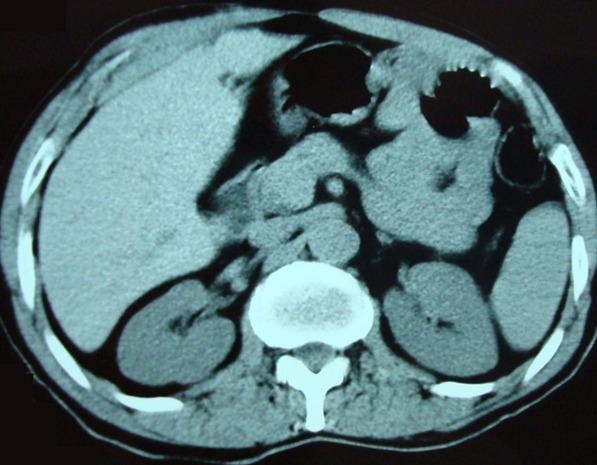
Coupe axiale tomodensitométrique montrant l’absence de vésicule biliaire

Patient âgé de 64 ans sans antécédents pathologiques, présentant depuis 2 mois des douleurs de l’hypochondre droit paroxystiques, à type de pesanteur sans rapport avec la prise alimentaire.Le bilan biologique est normal ; en particulier pas de cholestase, pas de cytolyse et absence d’hyperleucocytose. L’échographie abdominale objective un lit vésiculaire siège d’un écho dense avec cône d’ombre postérieur mesurant 13 mm de largeur en rapport avec une vésicule scléroatrophique lithiasique. Il n’y a pas de dilatation des voies biliaires intra et extra-hépatiques. On n’a pas fait de TDM. Vu l’aspect de vésicule scléroatrophique on sursoit à la cholécystectomie par voie coelioscopique et on indique une cholécystectomie par voie sous-costale droite. En per-opératoire, le pédicule hépatique est bien individualisé sans pouvoir visualiser la vésicule biliaire dans sa position habituelle, ainsi qu’après examen des sites d’ectopie vésiculaire (Figure 5). La calcification sus décrite à l’échographie n’a pas été retrouvée. L’échographie peropératoire confirme l’absence de vésicule biliaire ectopique intra-hépatique et on n’a pas fait de cholangiographie peropératoire de façon délibérée. Au cours du suivi, le patient a bénéficié un mois après l’intervention d’un scanner abdominal confirmant l’agénésie de la vésicule biliaire (Figure 6). On n’a pas trouvé d’autres anomalies associées ni en peropératoire ni sur le scanner. Une Bili-IRM a été demandée mais le patient a été perdu de vue.

## Discussion

L’agénésie de la vésicule biliaire est une aberration embryologique extrêmement rare avec une incidence allant entre 0,01% et 0,075% (10 à 75 pour 100 000 habitants) [3, 4]. La prévalence est estimée entre 1 et 10 pour 10.000 personnes dans la population générale [5]. On ne dispose pas d’étude épidémiologique sur le sujet en Tunisie. Le sexe ratio est de 1 pour les cas découverts à l´autopsie [1], mais le ratio femmes-hommes est de 3:1 dans les études cliniques, probablement parce que la symptomatologie est similaire à celle de la lithiase biliaire, plus fréquente chez les femmes [6]. Pour nos cas, on a une femme, un homme et une enfant de 13 ans. Cette anomalie est secondaire à un défaut de développement in utero du bourgeon caudal du diverticule hépatique, qui apparaît vers le milieu de la 3^ème^ semaine de gestation à la partie distale de l’intestin antérieur. Il faut signaler que l’agénésie de la vésicule biliaire est généralement accompagnée par une agénésie du canal cystique [7], comme c’est le cas dans nos trois observations. Jusqu´en 2006, seuls 3 cas d´agénésie de la vésicule biliaire avec présence du canal cystique ont été rapportés [8]. La responsabilité de l’embryogenèse est corroborée par l’association fréquente de l’agénésie à des malformations congénitales.

Dans ce contexte, Bennion et al. [4] ont établi une classification qui permet de distinguer 3 groupes: *a) Groupe des anomalies fœtales multiples (12,9 %):* ces patients décèdent généralement dans la période périnatale de leurs autres malformations, l´absence de vésicule biliaire est uniquement constatée à l´autopsie. Les malformations cardio-vasculaires sont les plus communes, elles sont suivies par celles des systèmes gastro-intestinalet génito-urinaire. *b) Groupe asymptomatique (31,6 %):* ce groupe de patients sans vésicule biliaire est découvert le plus souvent à l´autopsie ou au cours d´une intervention effectuée dans le but d´un autre diagnostic. Aucun de ces patients ne présente la moindre symptomatologie biliaire. *c) Groupe avec manifestations cliniques, forme symptomatique (55,6 %):* ce groupe correspond à la tranche d´âge de 40 à 50 ans, habituellement sans autres anomalies congénitales 2 de nos 3 observations correspondent à ce groupe.

Quand l’absence congénitale de la vésicule biliaire est symptomatique, les patients peuvent présenter des douleurs biliaires [8] et une perturbation du bilan hépatique, anomalies qui sont classées actuellement sous le terme de dysfonction du sphincter d’Oddi. La DSO est basée sur la classification de Milwaukee [9]qui est une classification clinico-biologique. Celle-ci comprend 4 critères: a) douleur biliaire; b) élévation des ASAT ou des phosphatases alcalines à plus de deux fois la normale; c) retard d’évacuation du produit de contraste après cholangiographie rétrograde supérieur à 45 minutes; d) diamètre de la voie biliaire supérieur à 12mm. Ce qui permet de distinguer trois types : type I (critères a + b + c + d), type II (a + b ou c ou d) et type III (a seul).

En pratique, cette classification permet donc d’apprécier globalement la probabilité de DSO. Ensuite, un test de confirmation est réalisé soit par manométrie biliaire, soit par scintigraphie biliaire. Les manifestations cliniques de l’agénésie vésiculaire peuvent être aussi à type d’ictère: en rapport avec une lithiase de la voie biliaire principale associée, laquelle est observée chez 20 à 50 % des patients présentant une agénésie [10]. L’association à une pancréatite aiguë a été décrite dans un seul cas dans la littérature [11], le deuxième cas serait celui de notre 2^ème^ observation. Comme autre conséquence physiopathologique de l’agénésie, on peut trouver une dilatation du cholédoque qui imite une vésicule biliaire rétrécie [4] pour compenser l’absence de vésicule en prenant la fonction de stockage de la bile. Le diagnostic préopératoire de l’agénésie vésiculaire n’est pas toujours simple à établir à cause de la rareté de cette anomalie et de l’échec des moyens d’imagerie hépatobiliaire d’atteindre une sensibilité de 100%. L’échographie est l’examen initial de référence pour l’exploration de la pathologie biliaire [12]. Sa sensibilité est de 95% dans le diagnostic de lithiase vésiculaire [10].

Dans les cas confirmés d’agénésie de la vésicule biliaire, le compte rendu d’échographie souvent rapporté par le radiologue est une vésicule scléroatrophique lithiasique; comme le cas de deux de nos observations. Ces faux positifs peuvent être expliqués par l’interposition d’anses grêles dans la fossette vésiculaire [13], l’interposition de plis péritonéaux périportaux [12] ou encore par des foyers de calcifications hépatiques [14]. Lanon visualisation d’une vésicule biliaire à l’échographie et au scanner nécessite la confirmation du diagnostic d’agénésie par la pratique d’une CPRM.

Actuellement la Bili-IRM est le gold-standard pour détecter une agénésie de la vésicule biliaire [8, 15]. C’est un examen d’imagerie non invasif qui permet de retrouver une vésicule biliaire dans un site ectopique (dans le ligament falciforme, le petit épiploon, au niveau du pancréas, derrière le duodénum, dans la paroi digestive pylorique, voire en intrahépatique), et en l’absence de vésicule retrouvée, de faire le diagnostic préopératoire correct de l’agénésie. La démarche thérapeutique diffère selon la circonstance de découverte (en pré ou en per-opératoire); selon l’association ou non à une lithiase de la voie biliaire principale et selon l’existence ou non d’une dysfonction du sphincter d’Oddi.

**Figure 7:**
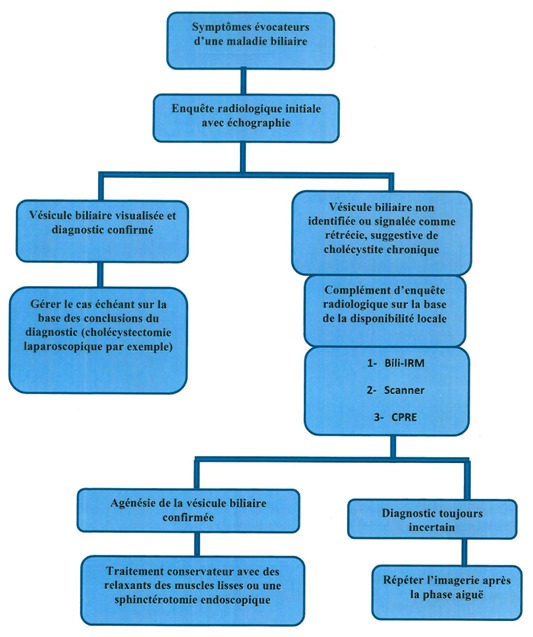
Algorithme décisionnel devant une agénésie de la vésicule biliaire

Concernant la découverte en préopératoire, ce sont des patients qui consultent pour des douleurs biliaires et à l’échographie, la vésicule est non identifiée ou bien signalée comme scléroatrophique. Dans ce cas, un complément d’exploration radiologique est nécessaire pour trancher entre une véritable vésicule scléroatrophique et une agénésie vésiculaire. Au mieux, une Bili-IRM doit être effectuée afin de poser le diagnostic avec certitude et dans ce cas il n’y a aucune indication chirurgicale (Figure 7).

Pour les agénésies de diagnostic peropératoire, ce sont des patients qui ont été opérés à tort pour lithiase vésiculaire [15, 16]. Ces malades peuvent être opérés soit par voie classique, soit par voie cœlioscopique. Au cours de l’intervention, on recherchera au mieux les différents sites ectopiques de la vésicule biliaireen s’aidant d’une échographie per opératoire si elle est disponible [14]. Par contre, on ne voit pas l’intérêt d’une conversion dans ce but. La cholangiographie peropératoire par ponction du cholédoque ne sera pas non plus effectuée de façon délibérée. En effet, il y a un risque élevé de blessure des voies biliaires ou de la veine porte au cours de la cholangiographie [17] alors que sa nécessité est remise en question. Dans tous les cas, on doit compléter par une bili-IRM en post opératoire pour confirmer le diagnostic [18]. Concernant les patients asymptomatiques, aucun traitement n’est requis.

Chez les patients qui présentent des douleurs biliaires en rapport avec une dysfonction du sphincter d’Oddi, le traitement comporte soit un traitement médical représenté essentiellement par les relaxants musculaires lisses utilisés pour soulager l’inconfort [19], soit la sphinctérotomie endoscopique qui est préconisée essentiellement pour les patients appartenant au type I de la classification de Milwaukee. Dans le cas d’agénésie associée à une lithiase cholédocienne, la conduite à tenir est la même qu’en cas de lithiase résiduelle, à savoir la sphinctérotomie endoscopique en 1ère intention et la chirurgie en cas de gros calcul avec impossibilité d’extraction.

## Conclusion

L'agénésie de la vésicule biliaire est une affection très rare, due à une aberration du développement embryologique. Elle peut être associée à d’autres anomalies congénitales, mais elle est le plus souvent découverte isolée à l’âge adulte. Elle doit être évoquée devant l'absence de visualisation de vésicule à l'échographie ou le plus souvent devant un aspect scléroatrophique. La bili-IRM permet de confirmer le diagnostic avec certitude et d’éviter une intervention chirurgicale qui pourrait faire courir des risques inutiles au patient. Il n’existe pas à proprement parler de traitement de l’agénésie vésiculaire mais celui des lésions associées qui en découlent, en particulier la lithiase cholédocienne et la dysfonction du sphincter d’Oddi.

## Conflits d’intérêts

Les auteurs ne déclarent aucun conflit d’intérêts.
